# Supplementary material for: Influence of Sucrose and Activated Charcoal on Phytochemistry and Vegetative Growth in Zephyranthes irwiniana (Ravenna) Nic. García (Amaryllidaceae)
Source: Plants (Basel). 2024 Feb 20;13(5):569. doi: 10.3390/plants13050569 (PMC10935178; doi:10.3390/plants13050569)
Supplement: Supplementary file 1 [file plants-13-00569-s001.zip › plants-2822661-supplementary.pdf]

**Supplementary Material:**

The chromatograms of the crude ethanolic extracts from each treatment (T1-T6 and GH) of leaves, bulbs and roots of *Zephyranthes irwiniana* is described below. α-terpinyl-acetate (blue column), ethyl linoleate (green column), lycorine (yellow column) and clionasterol (red column). X axis: Absolute Intensity (x $10^8$). Y axis: Retention time (min).

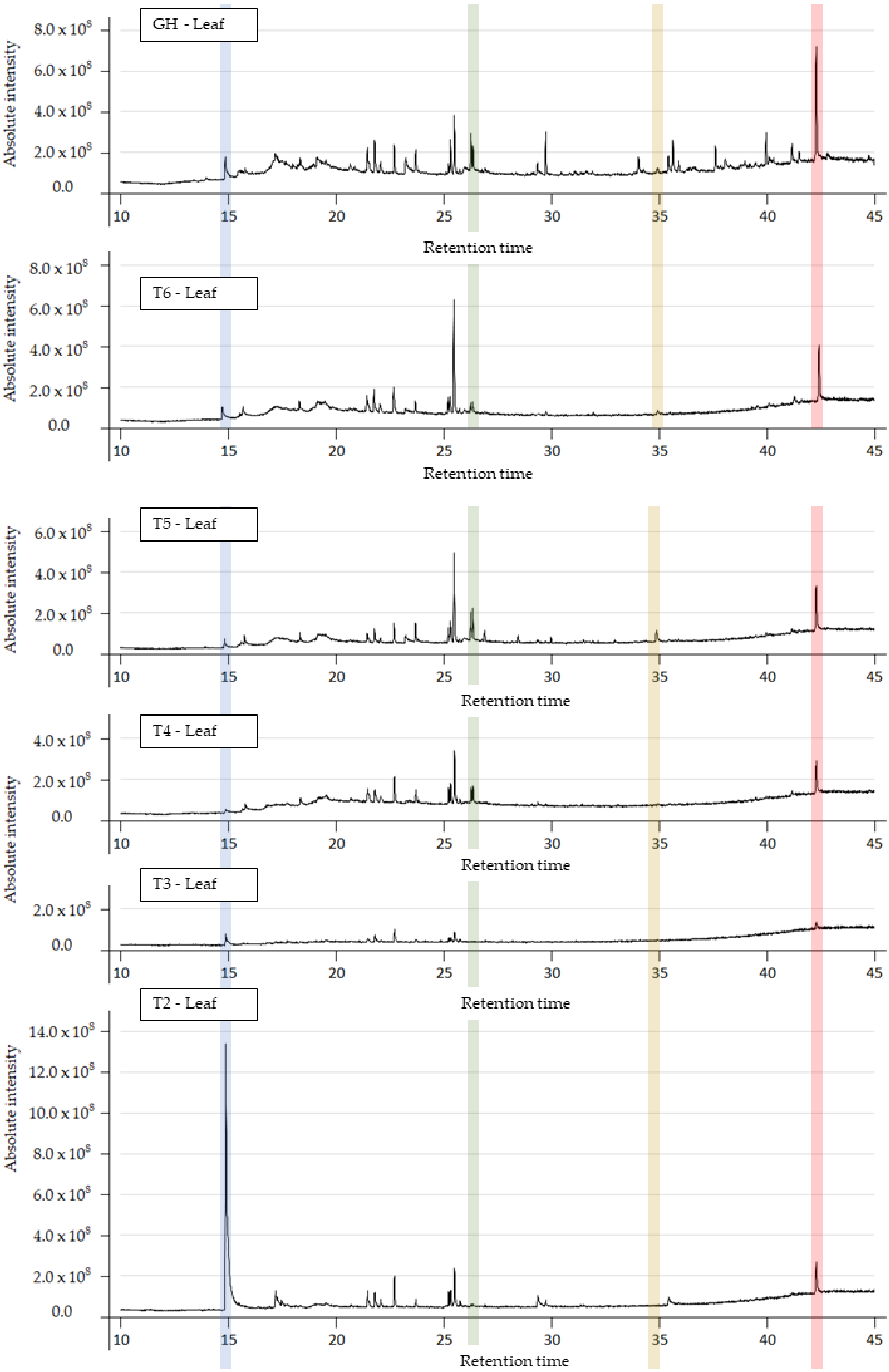

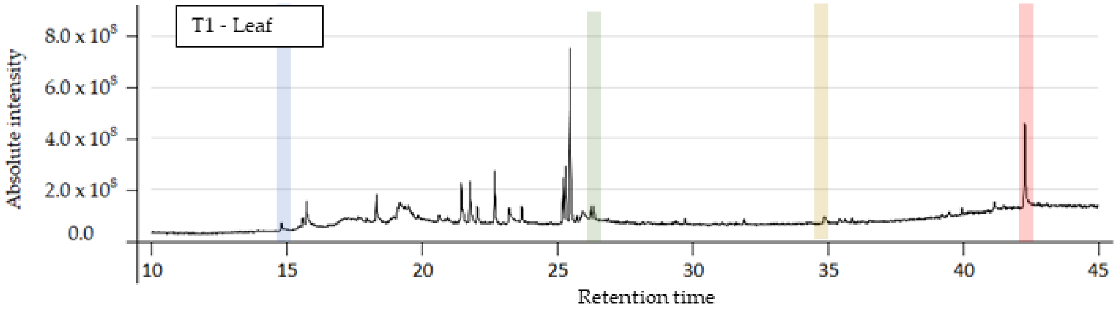


**Figure S1.** Chromatograms of the crude ethanolic extract from T1 to T6 treatments from leaves of *Zephyranthes irwiniana* using micropropagation and GH treatment from leaves of *Z. irwiniana* cultivated in greenhouse.

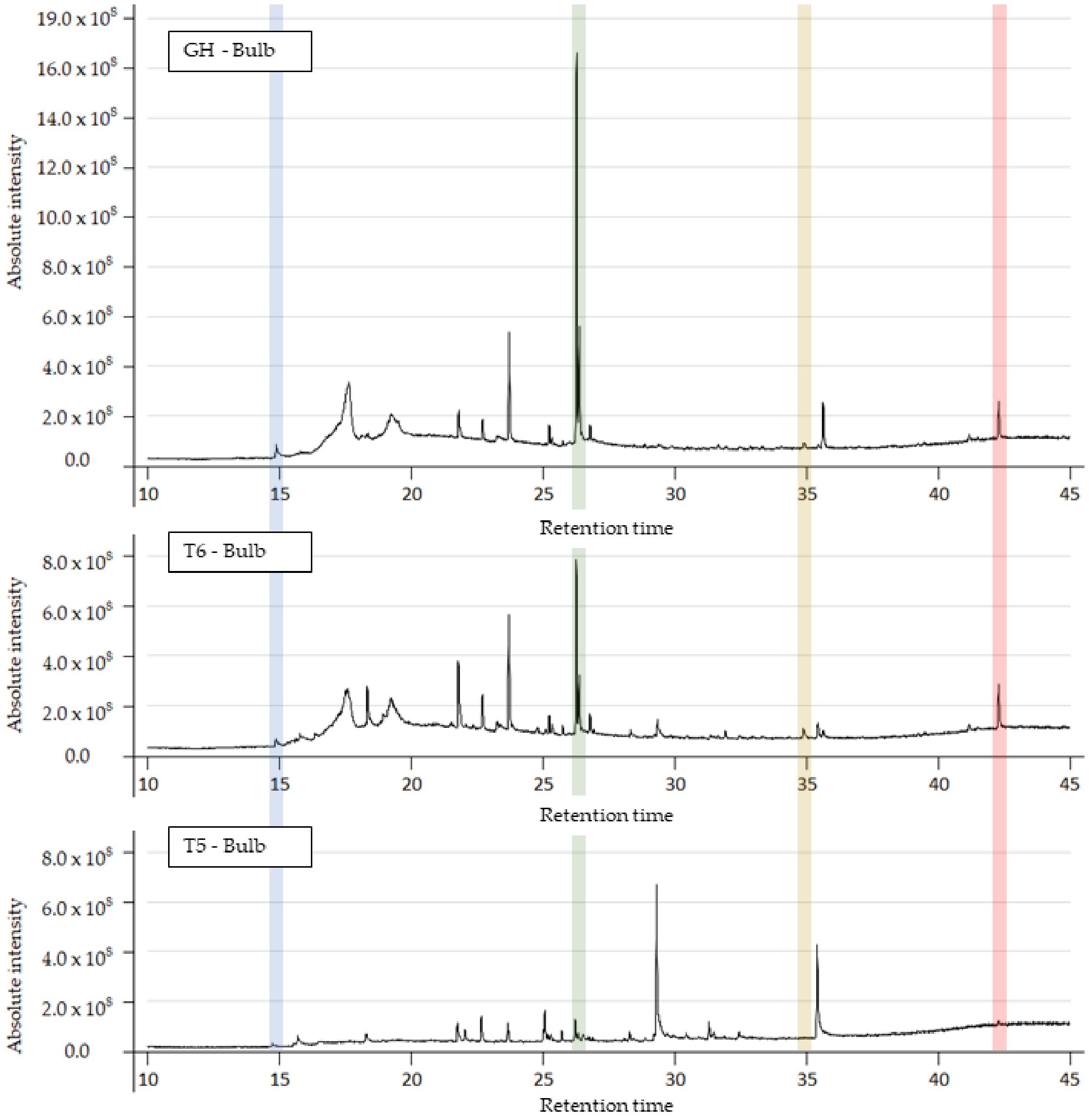

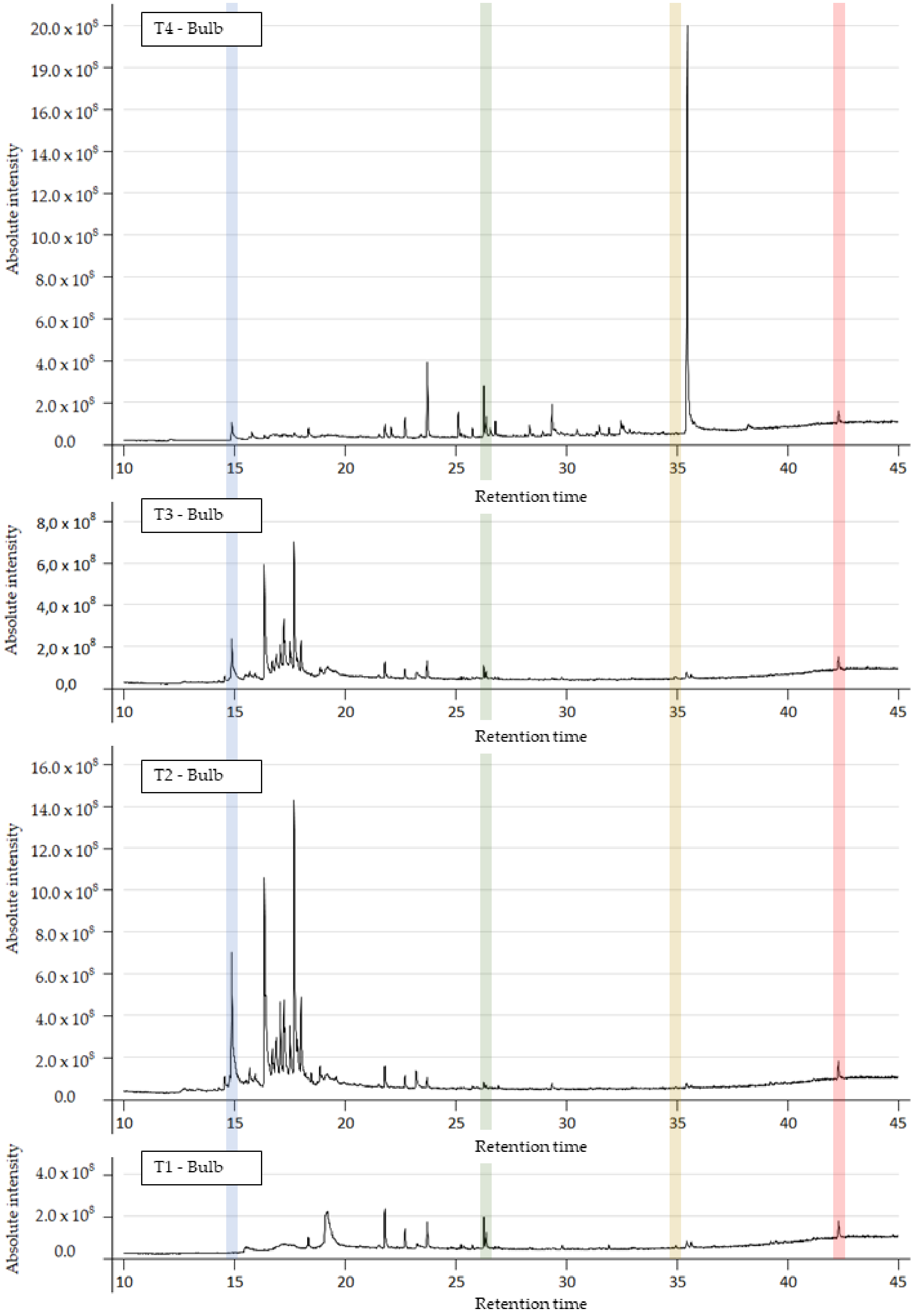


**Figure S2**: Chromatograms of the crude ethanolic extract from T1 to T6 treatments from bulbs of *Zephyranthes irwiniana* using micropropagation and GH treatment from bulbs of *Z. irwiniana* cultivated in greenhouse.

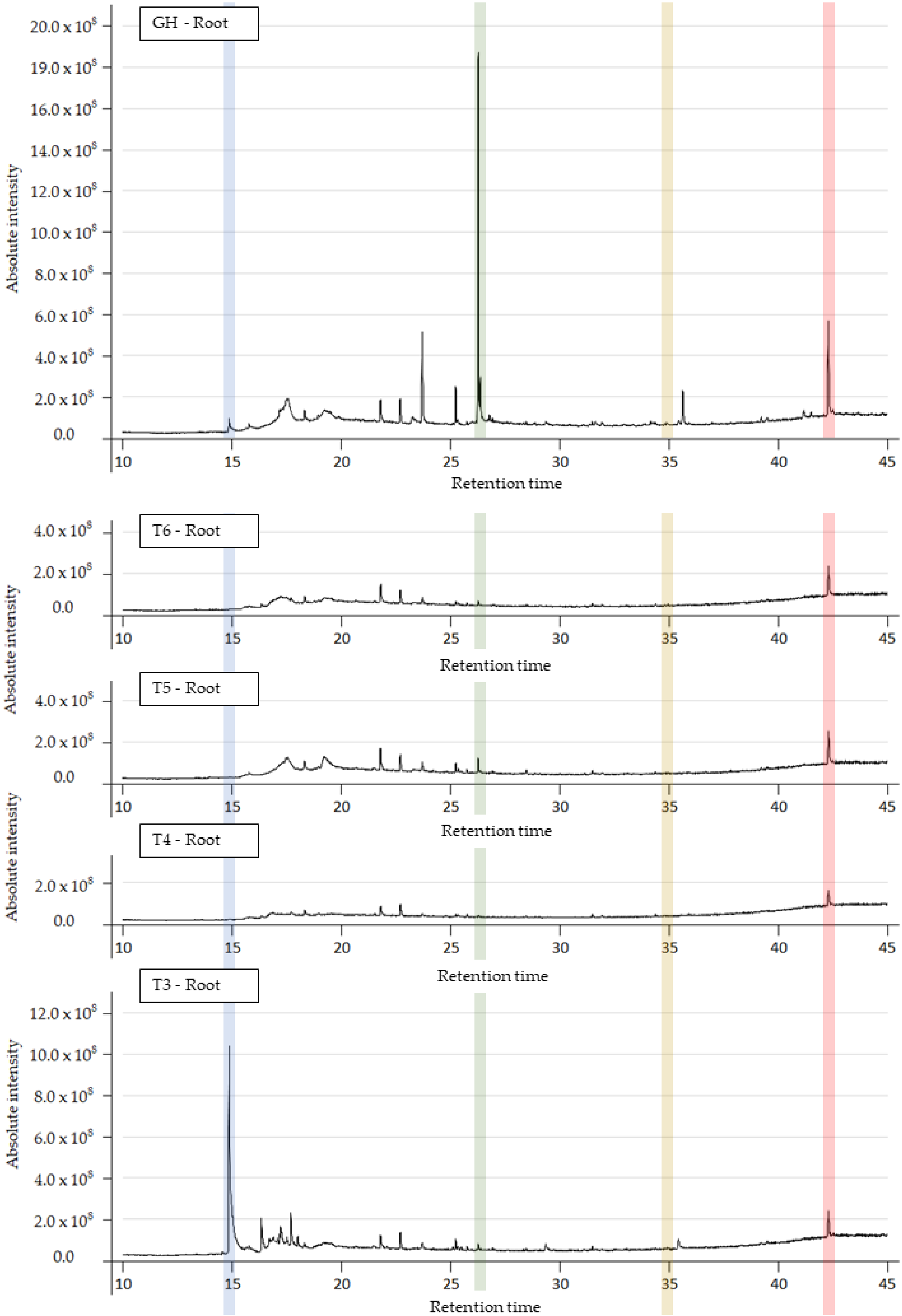
GH - Root
20.0 x $10^8$
19.0 x $10^8$
16.0 x $10^8$
14.0 x $10^8$
12.0 x $10^8$
10.0 x $10^8$
8.0 x $10^8$
6.0 x $10^8$
4.0 x $10^8$
2.0 x $10^8$
0.0
Absolute intensity
10
15
20
25
30
35
40
45
Retention time
T6 - Root
4.0 x $10^8$
2.0 x $10^8$
0.0
Absolute intensity
Retention time
T5 - Root
4.0 x $10^8$
2.0 x $10^8$
0.0
Retention time
T4 - Root
2.0 x $10^8$
0.0
Absolute intensity
Retention time
T3 - Root
12.0 x $10^8$
10.0 x $10^8$
8.0 x $10^8$
6.0 x $10^8$
4.0 x $10^8$
2.0 x $10^8$
0.0
Absolute intensity
Retention time

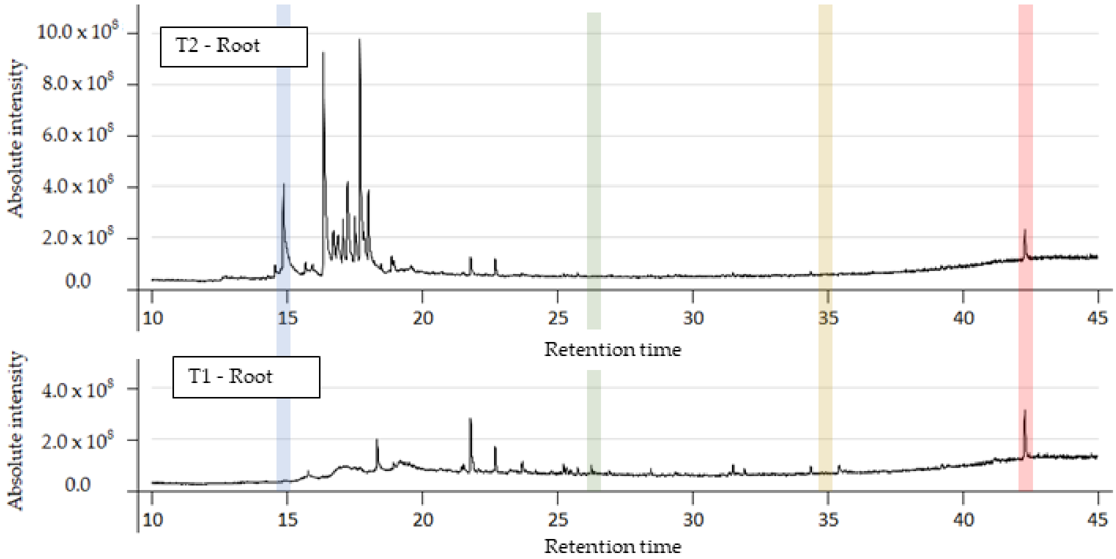


**Figure S3**: Chromatograms of the crude ethanolic extract from T1 to T6 treatments from roots of *Zephyranthes irwiniana* using micropropagation and GH treatment from roots of *Z. irwiniana* cultivated in greenhouse.
